# Surface Atomic Arrangement of Aluminum Ultra-Thin Layers Grown on Si(111)

**DOI:** 10.3390/nano13060970

**Published:** 2023-03-08

**Authors:** Inshad Jum’h, Husam H. Abu-Safe, Morgan E. Ware, I. A. Qattan, Ahmad Telfah, Carlos J. Tavares

**Affiliations:** 1 School of Basic Sciences and Humanities, German Jordanian University, Amman 11180, Jordan; 2 Department of Electrical Engineering, University of Arkansas, Fayetteville, AR 72701, USA; 3 Department of Physics, Khalifa University of Science and Technology, Abu Dhabi P.O. Box 127788, United Arab Emirates; 4 Leibniz-Institut für Analytische Wissenschaften-ISAS-e.V., 44139 Dortmund, Germany; 5 Nanotechnology Center (NTC), The University of Jordan, Amman 11942, Jordan; 6 Centre of Physics of Minho and Porto Universities (CF-UM-PT), University of Minho, 4804-533 Guimarães, Portugal

**Keywords:** C-Si(111), aluminum, MBE, surface structure, atmospheric oxidation, ellipsometry

## Abstract

Surface atomic arrangement and physical properties of aluminum ultrathin layers on c-Si(111)-7 × 7 and hydrogen-terminated c-Si(111)-1 × 1 surfaces deposited using molecular beam epitaxy were investigated. X-ray photoelectron spectroscopy spectra were collected in two configurations (take-off angle of 0° and 45°) to precisely determine the surface species. Moreover, 3D atomic force microscopy (AFM) images of the air-exposed samples were acquired to investigate the clustering formations in film structure. The deposition of the Al layers was monitored in situ using a reflection high-energy electron diffraction (RHEED) experiments to confirm the surface crystalline structure of the c-Si(111). The analysis of the RHEED patterns during the growth process suggests the settlement of aluminum atoms in Al(111)-1 × 1 clustered formations on both types of surfaces. The surface electrical conductivity in both configurations was tested against atmospheric oxidation. The results indicate differences in conductivity based on the formation of various alloys on the surface.

## 1. Introduction

Aluminum (Al) thin layers epitaxially grown on crystalline substrates such as silicon wafers are of particular interest for surface-sensitive applications such as surface-enhanced Raman spectroscopy (SERS) [1] and 2D material growth [2,3,4]. The thin layers serve as a scalable plasmonic platform in the SERS applications, as well as a growing template for building technologically important 2D materials, such as silicene [4,5]. Knowing the details of the surface structure and its crystalline arrangement is critical for the deposition of ultrathin metallic films, especially if protection against atmospheric oxidation is required. The metallic surface is harmed by atmospheric exposure, which causes partial amorphization and severely limits its functionality [6,7,8,9,10,11,12]. This affects surface electrical conductivity and controls the current efficiency in many applications [12,13,14]. To overcome this problem, it has been shown that surface restructuring of c-Si wafers can inhibit oxidation due to different adsorption mechanisms of oxygen on the surface [15,16,17,18]. Therefore, it is essential to study the conductivity of metallic ultrathin thin films grown on different crystalline arrangements of the c-Si surface. The corrosion degradation of functional layers on the surface caused by atmospheric air exposure is of particular interest.

The current study demonstrates the fabrication of ultrathin Al layers epitaxially grown on c-Si(111) surfaces with different surface arrangements, namely 7 × 7 reconstruction and hydrogen-terminated 1 × 1 surfaces. The deposition of the Al layers was monitored in situ using a reflection high-energy electron diffraction (RHEED) apparatus to confirm the surface crystalline structure of the c-Si(111) before and during the Al growth. Liu et al. have shown that monolayers (ML) of Al (here, one monolayer refers to the atomic density of an ideal one-atom-thick sheet) on c-Si(111)-7 × 7 exhibits remarkable stability at −128 °C and suffer atmospheric corrosions and amorphization at room temperature [19,20,21]. As a result, maintaining the metallicity of these layers in different environments is critical to ensure functional behavior. In this study, the deposited Al layers were tested against atmospheric oxidation by simply exposing them to ambient air at room temperature. We established for the first time a comparison between the surface conductivity of Si(111)-7 × 7 and hydrogen terminated Si(111)-1 × 1 surfaces with Al(111) ultrathin layer deposition. The results indicate a substantial difference in this regard based on the difference of amorphization caused by atmospheric air. The formation of Al-Si oxides on the rearranged surfaces was revealed by X-ray photoelectron spectroscopy (XPS) and spectral ellipsometry (SE). The intensity of the O 1s core line peaks was analyzed and found to be dependent on the surface arrangements. The thicknesses and optical properties of the Al-Si oxides system were determined through ellipsometry analysis. The electrical conductivity of the surface in both surface arrangements was measured by two probe I-V measurements. 

## 2. Materials and Methods

### 2.1. Sample Preparation

**Figure 1:**
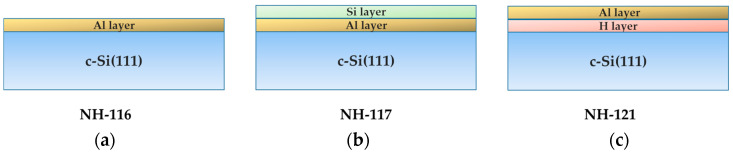
Schematics and naming of the fabricated samples: (**a**) NH-116 consisting of a monolayer of Al; (**b**) NH-117 consisting of monolayers of Si/Al; (**c**) NH-121 consisting of monolayers of Al/H.

Al layers were grown on 1 × 1 cm^2^ c-Si(111) wafers (p-type, 0.01–0.02 Ωm) in a molecular beam epitaxy (MBE) system equipped with a reflection high-energy electron diffraction (RHEED) apparatus with a base pressure of 5 × 10^−^^11^ mbar. The wafers were cleaned according to a standard set of wafer cleaning steps (RCA) [22,23,24]. Additionally, the native oxide was removed by dipping the wafers in a diluted (10%) HF solution for 15 s. Following cleaning, the wafers were immediately placed into the MBE loading compartment before being transferred to the deposition chamber. Three sets of Al samples were prepared on these substrates in three different surface configurations, as outlined in the scheme illustrated in Figure 1. 

In the first set, prior to the Al layers deposition, the temperature of the silicon substrate was ramped up to 900 °C to remove hydrogen, fluorine, and any residual surface oxide on the c-Si wafer. At this temperature, the Si(111)-7 × 7 surface reconstruction is established [25,26]. The aluminum growth in these samples was prepared in three steps according to the method adopted by Jiang [27]. For this configuration the first step was to deposit 0.33 monolayers (ML) of Al on the 7 × 7 surface at 600 °C with a rate of 0.0167 ML/s. In the second step, the samples were annealed for one minute at 650 °C to form an intermediate phase known as Si(111)-√3 × √3-Al [28,29]. The third step was to deposit 0.82 ML of Al with a lower temperature of 400 °C [27,30]. These samples formed the first set and were designated as NH-116. The second set of samples (named NH-117) was prepared in the same manner as NH-116, but an additional 0.60 ML of silicon on top of the prepared Al layer was deposited at a rate of 2.8 × 10^−^^5^ ML/s while keeping the substrate at 400 °C. 

The purpose of this layer was to form a surface protection layer for Al. The third set of samples (named NH-121) was prepared on silicon wafers without any thermal treatment prior to the growth process. Because of the HF dipping, the hydrogen-terminated c-Si(111) surface was maintained during the Al deposition by keeping the c-Si wafer at room temperature. In this case, the Al layers were expected to grow on the c-Si(111)-1 × 1 passivated surface through a hydrogen-assisted monolayer growth mechanism [31].

The growth process was monitored by RHEED live imaging operating at 16 keV electron beam, and the resulting images of the electron patterns were recorded. The samples were taken from the MBE system and stored in the lab under ambient conditions (atmospheric air pressure, 50–70% humidity at room temperature).

### 2.2. Sample Characterization

The XPS analysis of the samples was carried out using an Axis HS spectrometer from Kratos Analytical, equipped with a polychromatic dual-anode Mg-Kα/Al-Kα X-ray source and a hemispherical electron energy analyzer, where the kinetic energy of the photoelectrons was detected with the analyzer set to the magnetic mode with a pass energy of 20 eV. The spectra were collected in the standard configuration with a zero-degree take-off angle. As the samples are composed of MLs on the Si(111) substrate, a second set of spectra was taken with a 45° take-off angle for an angle-resolved (AR) XPS study. By comparing the peak intensities of spectra collected at different take-off angles, it is possible to determine the surface species more accurately since the analysis depth depends on the take-off angle: a larger take-off angle means a smaller depth analysis and thus greater surface sensitivity. A steel wire was used to connect the samples to the sample holder. This steel wire also added electrical contact, reducing surface charging. The pressure in the analysis chamber was <1.0 × 10^−^^8^ mbar during the measurements. CasaXPS version 2.3.15 software package was used to analyze the data. A Shirley background correction was used, and the spectra were fitted with Gaussian–Lorentzian curves (symmetric 70% Lorentzian for F 1s, C 1s, and Al 2p, and asymmetric 100% Lorentzian for O 1s and Si 2p). The Si 2p spectra were fitted considering spin-orbit splitting with a doublet separation of 0.61 eV and the area of the Si 2p_1/2_ peak set to half that of the Si 2p_3/2_ peak. A single FWHM was used for each photoelectron line (C 1s, O 1s, F 1s, Si 2p, and Al 2p) [32,33,34]. Spin-orbit splitting was not considered when fitting the Al 2p spectra because the doublet separation is very small (~0.4 eV); hence, only a single Al 2p peak centred at ~73 eV was used. The Al K_α3_ and Al K_α4_ satellite peaks were not fitted. The peak positions were used to identify the species in the samples using the NIST database [35], and the peak areas were used to determine the relative concentration of each species. Atomic Force Microscopy (AFM) analyses performed with a Park Systems XE 100 using the XEP 1.8.9. Build4 measurement software. Gwyddion 2.56 software was used to process the data. The spectral ellipsometry (SE) measurements were performed using a variable angle spectroscopic ellipsometer (VASE) to acquire the Ψ and ∆ parameters. The measurements were performed in air at room temperature over the wavelength range 270–1000 nm in steps of 10 nm at incidence angles of 65°, 70°, and 75°. The data were analyzed using the CompleteEASE software (version 4.58) to determine the structural and optical properties of the films.

## 3. Results and Discussion

**Figure 2:**
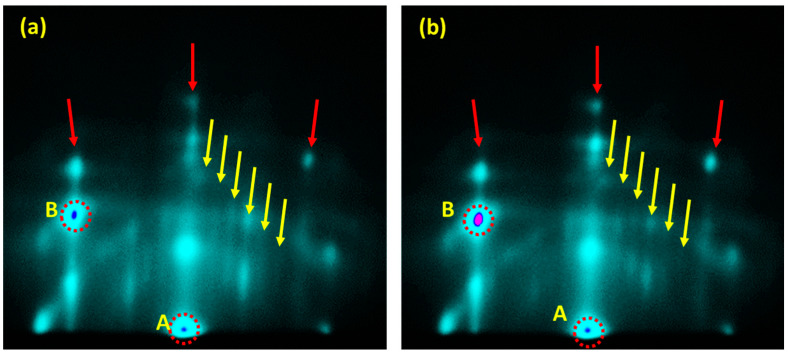
RHEED patterns (**a**) before the Al deposition and (**b**) after the completion of the Al layer. The red arrows indicate the 1 × 1 pattern of the c-S(111) wafer. The yellow arrows indicate the strikes of the 7 × 7 surface structure. The intensity of the A and B spots enhances as an Al(111)-1 × 1 layer is formed (as seen by comparing the B spot intensities in both figures).

Figure 2a shows the RHEED pattern of the Al films grown on the Si(111)-7 × 7 surface. The image was captured in the [2,3,4,5,6,7,8,9,10,11] azimuth direction after 2 h of annealing. The specular beam intensity indicated by spots A and B identifies the Si(111)-1 × 1 lattice originating from the bulk. These spots mark the 00 and 01 surface diffraction patterns, respectively. The streaky nature of these patterns suggests regions with flat surfaces. Figure 2b shows the diffraction pattern after the Al(111)-1 *×* 1 surface was established. The 7 × 7 streaks did not completely vanish and became blurry. This could be due to the high-temperature deposition (600 °C) at which the 7 × 7 surface was constructed. It is also worth noting that during the construction of the Al layer, the specular beam intensity of spot B increases significantly (indicated by the pink color in Figure 2b). This behavior is attributed to inelastic scattering of the reflected electrons by the Al atoms settled at the T4 sites. These are preferred sites for Al atoms on the c-Si(111)-7 × 7 surface [28].

**Figure 3:**
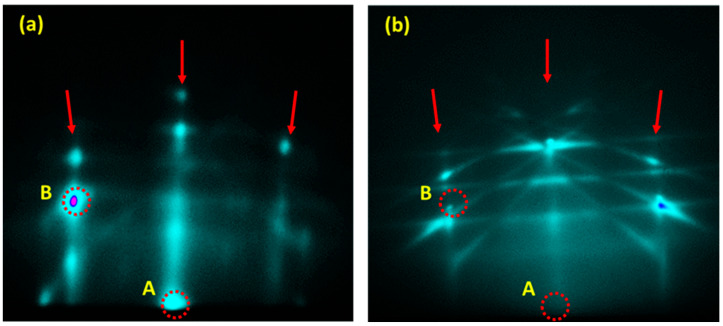
RHEED pattern of (**a**) the NH-117 samples after depositing 0.6 ML of Si atop the Al (111)-1 × 1 layer. The intensity of spot A is diminished. (**b**) NH-121 samples after forming the Al layer on the hydrogenated Si surface. Clear streaks and Kikuchi lines originated from a flat epitaxial Al (111)-1 × 1 surface are observed. In this case, no intensities are observed for spots A and B.

The intensity of the beams at the A and B spots is primarily determined by the first-order constructive interference of the diffracted beams. The increase in the intensity of these spots (as can be seen for spot B) indicates the Al deposition at these locations. Figure 3a shows the RHEED pattern for the NH-117 samples, where the Al deposition was followed by an additional 0.60 ML of silicon. The 7 × 7 streaks in this sample vanish, and the evolved pattern indicates a structure similar to the Si(111)-1 × 1 surface. This means that the new silicon surface on top of the Al formation mimics the Si(111)-1 × 1 surface underneath the Al layer. For the (111) surface, Al(111) and Si(111) have interatomic distances equal to $a_{Al}=2.86\;Å$ and $a_{Si}=3.84\;Å$, respectively. A close examination of these constants on the surface reveals that because 2.86/3.84 is almost 3/4, a 3 × 3 unit cell of Si(111)-1 × 1 closely matches the 4 × 4 unit cell of Al(111)-1 × 1, with a isostructural mismatch of 0.4%. As a result, one can anticipate epitaxial growth of Al(111) on Si(111) with minor interfacial strain [19,36].

**Figure 4:**
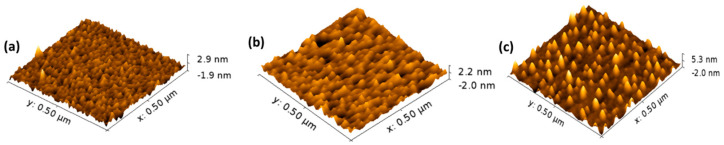
3D AFM images of the fabricated samples (**a**) NH-116 (**b**) NH-117 and (**c**) NH-121.

For the case of the silicon-hydrogen-terminated surfaces, Al was directly deposited on the hydrogen-terminated Si surface (samples NH-121). Figure 3b shows the RHEED pattern after Al(111)-1 × 1 is formed at room temperature. The A and B spots have very low intensity, indicating diffuse reflection from the Al layer due to its patchy structure. Nonetheless, due to a bulk diffraction effect, the surface was characterized by a well-defined (1 × 1) streaky RHEED pattern and high intensity Kikuchi lines. The RHEED pattern of this set indicates that the Al(111)-1 × 1 layer was grown epitaxially on the hydrogen-terminated Si(111)-1 × 1 surface. As a result, any generated stress during epitaxial growth was released by hydrogen-assisted deposition [28]. It can be inferred that the energy of the Al atoms in the presence of hydrogen has sufficient mobility to difuse and form 1 × 1 surface that mimic the underneath arrangement of the 1 × 1 silicon surface [28]. Figure 4 shows the 3D AFM images of the air-exposed samples. The NH-116 and NH-117 samples (the 7 × 7 samples) reveal adjacent clustered formations in continuous film structure. These features were more noticeable in sample NH-117 due to the additional 0.6 ML Si layer. On the other hand, the NH-121 sample shows isolated clusters with bigger size formations. Clustering of the deposited layers is caused by amorphization due to the presence of oxides in the films [37,38].

Table 1 shows the elemental composition of the air-exposed samples based on the XPS results. Increasing the take-off angle, and thus surface sensitivity, reduced the silicon 2p core line intensity while increasing the presence from adventitious carbon in the C 1s core line and fluorine concentrations (NH-121). This indicates that carbon and fluorine are most likely impurities adsorbed to the surface. The oxygen concentration changes as well, but in an inconsistent manner: it remains constant for NH-116, increases for NH-117, and decreases for NH-121. However, it still increases relative to the silicon concentration ([O]/[Si] increases), indicating that oxygen is also present near the surface of the sample. For Al, the behavior of both [Al] and [Al]/[Si] is inconsistent: both decrease for NH-116, both increase for NH-117, and [Al] remains relatively constant while [Al]/[Si] increases for NH-121. The intensity of the Al 2p core line is very low in all three samples, implying that the peak is barely detectable. Consequently, it has a low signal-to-noise ratio, which introduces uncertainty in the calculated peak area and thus [Al]. In addition, the Al layer is very thin, only a single monolayer, i.e., about one atom thick, which is too low for an accurate AR-XPS analysis. Thus, the most likely cause of these unexpected variations in [Al] is an experimental error caused by the samples’ overall low concentration of Al.

**Figure 5:**
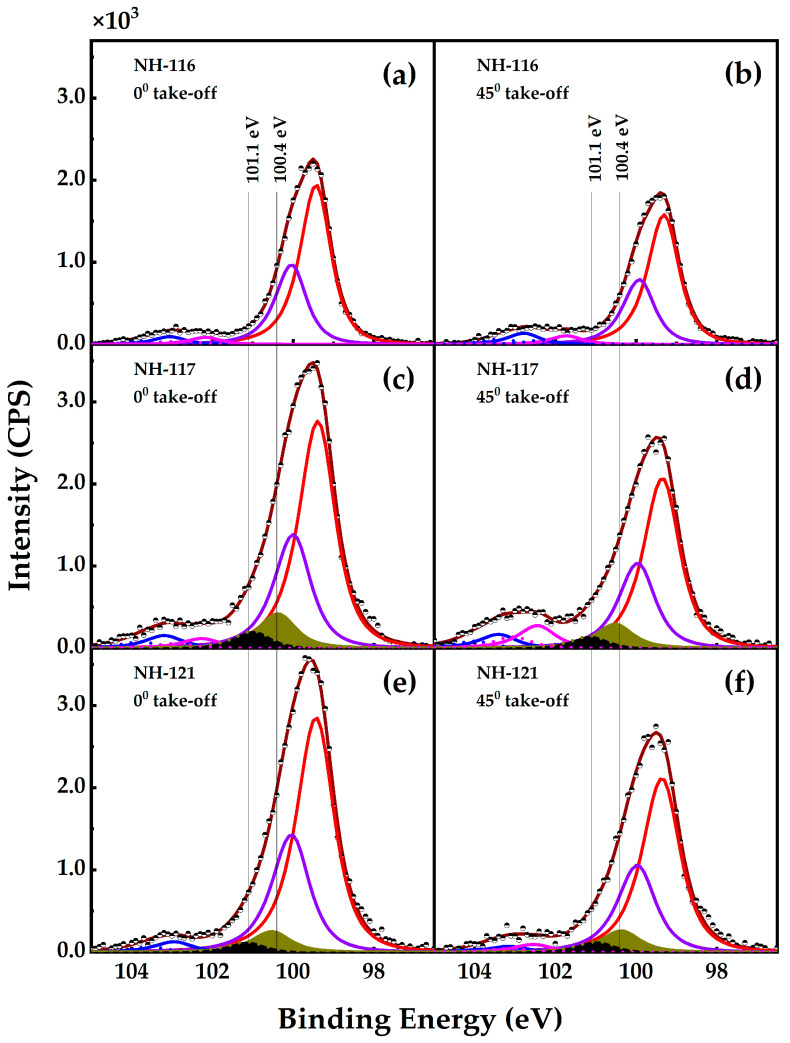
(**a**–**f**) Si 2p core line XPS spectra of the samples measured at take-off angles of 00 and 450. The contributions from different Si species in the samples (Table 2) are shown (#1 = red, #2 = blue, #3 = dark yellow, #4 = violet). Dots represent the experimental spectra, and the brown line represents the fit to the four contributions. The contribution seen as a black band in (**c**–**f**) is assigned to Si-O bonds from the exposed Si substrate.

The peak fitting of the Si 2p core line spectra indicates the presence of four different types of silicon bonds near the sample surface. The assignments of these peaks are given in Table 2. The data for the spin-orbit splitting were obtained with a doublet separation of 0.61 eV and the area of the Si 2p_1/2_ peak set to half of that of the Si 2p_3/2_ peak. Figure 5 shows the Si 2p XPS spectra of the samples measured at the two take-off angles, 0° and 45°.

The angle-resolved study shows that the intensity of the elemental silicon peaks decreases relative to the other peaks, indicating that elemental silicon (species #1) constitutes the bulk of the sample, whereas the other species are closer to the surface. It is noticed here that species #3 (namely aluminum silicide) is present only in NH-117 and NH-121 samples. The presence of this alloy in the NH-117 could be explained in terms of the spontaneous interaction of silicon with the Al, as the deposited silicon reaches the surface at an elevated temperature (400 °C) [39,40]. The silicide formations in the NH-121 samples are due to the reductions in the incoherence stress, which facilitate the interaction of silicon and aluminum atoms at the surface via the hydrogen assisted growth process as mentioned earlier [31]. The peaks at 101.1 and 100.4 eV appear only in NH-117 and NH-121. These peaks are characteristic of un-oxidized AlSi in the samples.

As previously mentioned, the Al 2p core line spectrum has a very low intensity. Therefore, it has only been fitted with a single broad Gaussian–Lorentzian curve. The Al 2p peak positions are at 75.5 eV for all produced samples (Table 3).

The O 1s core line spectra (not shown) are composed of a single broad asymmetric peak at 532.5 eV. It is a composite of unresolved peaks from various oxygen species. The oxygen species are SiO (532.5 eV), SiO_2_ (532.9 ± 0.4 eV), AlxSiyOz (peak at 532.0 ± 0.5 eV), and Al_2_O_3_ (531.2 ± 0.8 eV) [41,42]. Small amounts of organic oxygen (C=O peak at ~532.3 eV, C-O peak at ~533.0 eV) were also present.

The peak fitting of the C 1s core line reveals five different types of carbon species, whereby the C-F peak appearing only in the C 1s spectrum of sample NH-121. These carbon species are impurities adsorbed on the sample surface. The peak positions and their assignments are provided in Table 4.

Only sample NH-121 contains fluorine. Two peaks representing two different fluoride species were fitted to the experimental spectra. The high-intensity peak at 689.3 ± 0.6 eV binding energy is assigned to –CFx species, and the low-intensity peak at 686.1 ± 0.3 eV is assigned to AlF_3_·3H_2_O [42]. These fluorides are impurities that have been adsorbed to the surface of the sample.

For the ellipsometry analysis, the deposited samples were presented with a model of one layer that included several composite materials. The calculated thicknesses and material concentrations (at.%) are included in Table 5.

The concentration of pure Al in the films was calculated from the XPS refinements (Table 1) for a 45° take-off angle (since it has higher penetration depth), and found to be 0.103, 0.113 and 0.006 at.% for the NH-116, NH-117 and NH-121 samples, respectively. The metallic inclusions were protected by the various oxides and formed an interconnected network that promoted conductivity on the surface of the fabricated films.

**Figure 6:**
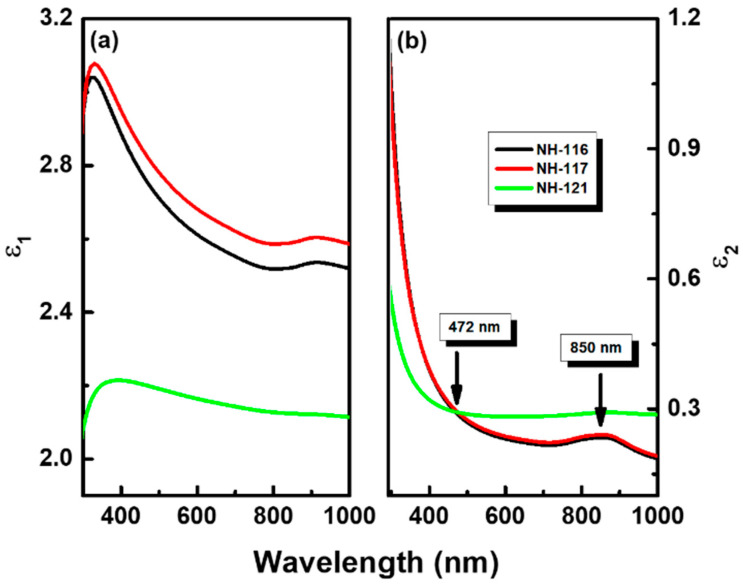
Spectral behavior of the (**a**) real (ε_1_) and (**b**) imaginary (ε_2_) parts of the complex dielectric constant ε of the films. The peaks in the imaginary part of the spectrum at 850 nm are related to the interband transition in Al.

Figure 6 shows the spectral behavior of the real ($\epsilon_{1}$) and imaginary ($\epsilon_{21}$) parts of the complex dielectric constant ($\epsilon =\epsilon_{1}+i\epsilon_{2}$) obtained for the films on the three surface arrangements. Based on the I-V measurements, one can conclude that the difference in optical properties between the three surfaces is linked to the presence of different percentages of metallic Al in the films. The ε_1_ spectrum shows a higher dielectric response across the measured spectra for the 7 × 7 samples (NH-116 and NH-117). On the other hand, the imaginary part (ε_2_) spectrum of the hydrogen-terminated surface (NH-121) shows a higher response beyond 472 nm in comparison to NH-116 and NH-117. This behavior results from the films microstructure with many Al clusters. The peak at 850 nm is related to the interband transition of pure aluminum. The ellipsometry measurements were repeated on a regular basis for several weeks, and the results consistently indicated the stability of these formations on the surface.

**Figure 7:**
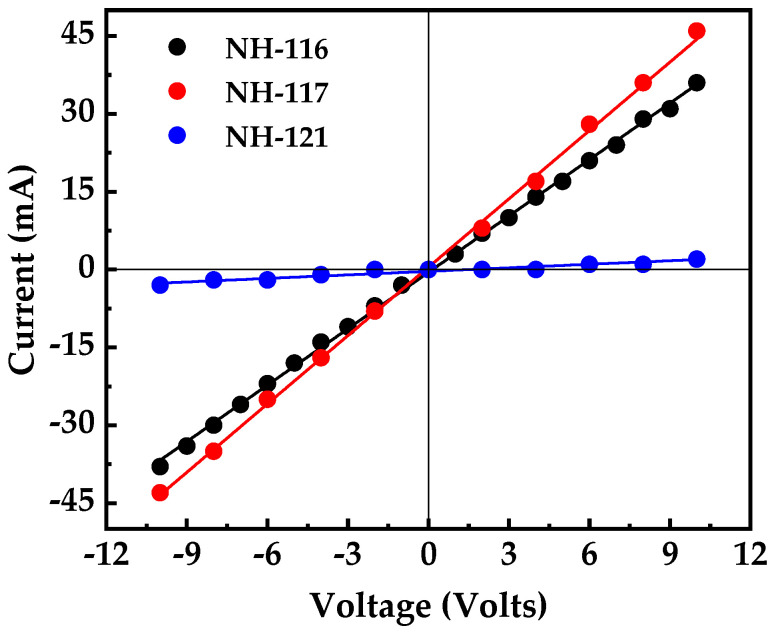
I–V measurements of the fabricated films.

The conductivity of the Al layers was obtained through simple two-probe I–V measurements, and the results are shown in Figure 7. The fitting of the data points for each sample indicated that the exposed samples are conductive in both configurations. The generated current was greater for samples deposited on the 7 × 7 surface. The aluminum on these surfaces formed silicide patched clusters, the increase in conductivity is attributed to the formation of these clusters, as evidenced from the AFM images and the following XPS discussion [39,40]. The silicide formed in the case of hydrogen terminated surface was included in isolated structures resulting in lower current conductivity compared to 7 × 7 samples. Therefore, it can be concluded that the surface reconstruction to accommodate higher surface conductivity is important and yield efficient current generation which is important for certain type of application such as photovoltaics and Schottky nanogenerator, etc. [43,44,45].

## 4. Conclusions

The current study demonstrates the fabrication of ultrathin Al layers epitaxially grown on c-Si (111) surfaces with different surface arrangements, namely 7 × 7 reconstruction and hydrogen-terminated surfaces. The reconstruction and Al growth on the 7 × 7 surface was performed in multistep process at elevated temperatures. On the other hand, the growth of Al on the hydrogen-terminated surface was carried out at room temperature. The analysis of RHEED patterns during the growth process suggests the settlement of aluminum atoms in Al(111)-1 × 1 clustered formations on both surfaces. The Al layers were tested against atmospheric oxidation by simply exposing them to ambient air at room temperature. The formation of Al-Si oxides on the rearranged surfaces was studied by combined XPS and spectral ellipsometry experiments. The detailed analysis of electric conductivity showed that he reconstructed silicon surfaces endure a higher atmospheric amorphization and establish a reliable conductivity in time. The Al layers in both arrangements have prospect as a material of choice for plasmonic applications and 2D material growth.

## Figures and Tables

**Table 1:** Elemental composition (at.%) of the air-exposed samples based on the XPS experiments.

	Take-Off Angle					
	NH-116		NH-117		NH-121	
Element	0°	45°	0°	45°	0°	45°
Si	37.6	30.3	38.3	31.3	39.5	28.3
Al	3.6	2.1	1.6	2.1	1.3	1.2
O	27.2	27.0	31.7	35.7	21.2	20.4
C	31.6	40.6	28.4	30.9	32.4	42.8
F	-	-	-	-	5.6	7.3

**Table 2:** Si 2p core line binding energies and their assignment for the different silicon species.

Si Species	Peak Position (eV)			Assignment
	NH-116	NH-117	NH-121	
#1	99.4	99.4	99.4	Elemental Silicon
	100.0	100.0	100.0	
#2	102.9	103.1	103.1	SiO_2_ or Al_x_Si_y_O_z_
	103.5	103.7	103.7	
#3	-	100.4	100.4	AlSi, SiC
	-	101.1	101.1	
#4	101.9	102.5	102.5	SiO or Al_x_Si_y_O_z_
	102.6	103.2	103.2	

**Table 3:** Al 2p peak positions and their assignments for the different aluminum species found in the samples.

Sample	Peak Position (eV)	Assignment
NH-116	75.5	Al_2_O_3_/Si
NH-117	75.5	Al_2_O_3_/Si
NH-121	75.5	Al_2_O_3_/Si

**Table 4:** The C 1s peak positions and their assignment for the different species found in the samples.

C 1s Peak	Peak Position (eV)			Assignment
	NH-116	NH-117	NH-121	
1	285.7	285.7	285.4	-CH_2_C(O)O
2	287.5	287.3	287.2	-C(O)-, CH_3_OH/Si
3	290.0	290.0	289.3	-C(O)O, CO_3_, CO_3_
4	283.5	283.6		SiC
5			292.0	-CF_8_

**Table 5:** Results of the ellipsometry analysis; MSE is the mean square error.

	MSE	Thickness (nm)	Voids %	Al%	SiO_2_%	Al_2_O_3_%
NH-116	2.87	2.70	43.2	4.9	26.8	25.1
NH-117	2.88	2.73	42.2	5.4	26.6	25.8
NH-121	2.43	3.38	25.2	0.5	29.3	45.0

## Data Availability

The datasets generated during and/or analyzed during the current study are available from the corresponding author upon reasonable request.
